# Trends and future directions in childhood obesity research in the Nordic countries: a scientometric review

**DOI:** 10.1093/eurpub/ckaf053

**Published:** 2025-04-15

**Authors:** Fereshteh Baygi, Kimiya Gohari, Shirin Djalalinia, Jeanette Reffstrup Christensen, Jesper Bo Nielsen, Jens Søndergaard

**Affiliations:** Research Unit of General Practice, Department of Public Health, University of Southern Denmark, Odense, Denmark; Department of Environmental Medicine and Climate Science, Icahn School of Medicine at Mount Sinai, New York, NY, United States; Deputy of Research & Technology, Ministry of Health and Medical Education, Tehran, Iran; Research Unit of General Practice, Department of Public Health, University of Southern Denmark, Odense, Denmark; Research Unit of General Practice, Department of Public Health, University of Southern Denmark, Odense, Denmark; Research Unit of General Practice, Department of Public Health, University of Southern Denmark, Odense, Denmark

## Abstract

In the Nordic countries, about one in five children aged 5–18 years live with overweight or obesity. This scientometric review analyses the patterns of childhood obesity research in the Nordic region to inform future strategic decisions for researchers and policymakers. Using VOSviewer (version 1.6.20), we conducted a visualization analysis of Nordic childhood obesity literature from Scopus, covering publication up to February 2024. Additionally, R version 4.4.0, and Microsoft Excel 2016 were used to support further analysis. The analysis included trends of scientific outputs, citations, patterns, collaboration network, and leading institutions. In the Nordic countries, 4123 documents were published from 1981 to 2024. A consistent increase was identified in collaborative studies since 1981. Sweden is playing a leading role in childhood obesity research. A strong partnership was noted between Danish and Swedish researchers, as well as between Finnish and Swedish researchers, with Sweden being a central hub of collaboration. The highly cited publications primarily focused on non-pharmacological public health interventions to reduce cardiovascular risk factors. Significant advancement has been achieved in understanding childhood obesity primarily focused on the filed medicine and nursing. Overweight, obesity, and metrics like body mass index have been extensively investigated, but no focus has been placed on medication as treatment. Despite the increasing research in this field, knowledge gaps exist in genetics, molecular biology, emerging pharmacological treatment as well as behavioral and social sciences. Future research should utilize the unique Nordic databases and advanced methods to improve understanding and inform effective public health interventions.

## Introduction

Over the past 4 decades, the prevalence of overweight and obesity has risen significantly on the global scale [1, 2]. In the Nordic countries, overweight and obesity impacts approximately one in five 5–18-years-old children [3–5], making it a significant public health threat. Previous studies have shown pre-school and childhood overweight, and obesity are linked to adolescents’ obesity and adverse health outcomes in adulthood [6, 7]. Therefore, we believe that continuous monitoring of studies in this field is needed to develop effective health promoting strategies to prevent overweight and obesity.

Our research exclusively centers on Nordic countries including Sweden, Finland, Iceland, Norway, and Denmark. These countries have comparable healthcare systems [3] and exhibit similar prevalence rates of overweight and obesity [3, 6]. The most recent World Health Organization (WHO) report on the topic states that the prevalence of overweight and obesity in the Nordic countries ranged from 25.2% to 30.9% among 5–9-years old children and from 22.9% to 27.0% among those aged 10–19 [6]. This report shows higher prevalence in specific age groups compared to overall estimate mentioned above [3–5]. Further, a newly released review among children from birth to 7-years old revealed that most of the interventions in the Nordic countries failed to produce positive long-term outcomes for obesity management [3]. There exists no similar study among school-age children in the Nordic countries. Hence, the aim of this study is to assess the patterns of scientific publications related to both prevention and treatment of obesity among school-age children in the Nordic countries. We believe that employing an analytical approach in scientific outputs within this field is crucial, as it can provide valuable insights into scientific progress and inform strategic developments for researchers and policymakers. This analytical perspective enables the implementation of prevention and management of programs informed by trend studies. This study addressed the following research questions:

1. What are the patterns of research on childhood overweight/obesity (e.g. research landscape, influential studies, key contributors, collaboration networks, focus areas) in the Nordic countries?
2. Which gaps exist in the current research, knowledge, and treatment options (e.g. anti-obesity medications) for childhood overweight/obesity in the Nordic countries?
3. What would be the priorities for future studies in this research area in the Nordic countries?

## Methods

### Study design

This study is a scientometric analysis of research papers on overweight/obesity among school-age children in the Nordic countries. This study was carried out without restrictions on the time of publication of scientific documents or the language of their publication.

### Eligibility criteria

The eligibility criteria included: (i) Only peer-reviewed original articles, and systematic reviews were considered. (ii) Research must specifically focus on childhood overweight/obesity- according to any parameter including BMI, BMI *z*-score, waist circumferences (WC), weight-to-height- ratio (WhtR), percent body fat- as a primary or secondary outcome. (iii) Research must be conducted in one of the Nordic countries (Sweden, Finland, Iceland, Norway, and Denmark).

### Data source

The Scopus database was selected as the most comprehensive source for searching scientific documents due to its wide range of topics, including interdisciplinary subjects [8]. It was used as a reliable source for citation reports as it indexes scientific documents with citation features [8]. Furthermore, Web of Science (WOS/ISI) citation database was searched and examined for its capability to analyze thematic fields of related research [8].

In addition to reviewing and analyzing the trends and specific features of articles as key indicator for knowledge production, this study focused on quantitative indicators such as article citations and external collaborations. To investigate the interactive network between researchers from various institutions and map the most frequent keywords used in published studies, a systematic and comprehensive search was conducted through PubMed, Embase and WOS/ISI, as well as snowball search for grey literature and reference lists from relevant reviews. Duplicate and unrelated items were removed, and the resulting comprehensive dataset was used as an input for the visualization software.

### Search strategy

The search strategy was designed by the research team and validated by an external scientific group including experts in public health and epidemiology, who had experience in obesity research. They reviewed the search strategy and Emtree terms such as “obesity,” “overweight,” and “anthropometric indexes,” to ensure comprehensiveness and accuracy. The Nordic countries’ names, as listed above, were identified according to Wikipedia. The search strategy is presented in Supplementary Table S1.

### Visualized and statistical analysis

VOSviewer (version 1.6.20) software was used to perform bibliometric visualization of publications [9]. In this study, VOSviewer was employed for co-occurrence, co-authorship, co-citation, and bibliographic coupling analyses. Additionally, R version 4.4.0 (R Core Team, Vienna, Austria) and Microsoft Excel 2016 (Microsoft Corporation, Redmond, WA) were used to support further analysis and data visualization.

## Results

### Study selection process

Supplementary Fig. S1 shows an overview of the study selection process. The Nordic countries published in total 4123 articles on this topic between the years 1981 and 2024. Following deduplication, 2938 articles remained. After the eligibility criteria were applied, 2441 studies were excluded. Out of the remaining 497 studies, 56 were excluded after the full-text review, leaving 441 research papers to be included in the analysis. Of the 441 studies, there were 434 research articles and seven review articles.

### Publication trends in the Nordic countries

**Figure 1.**
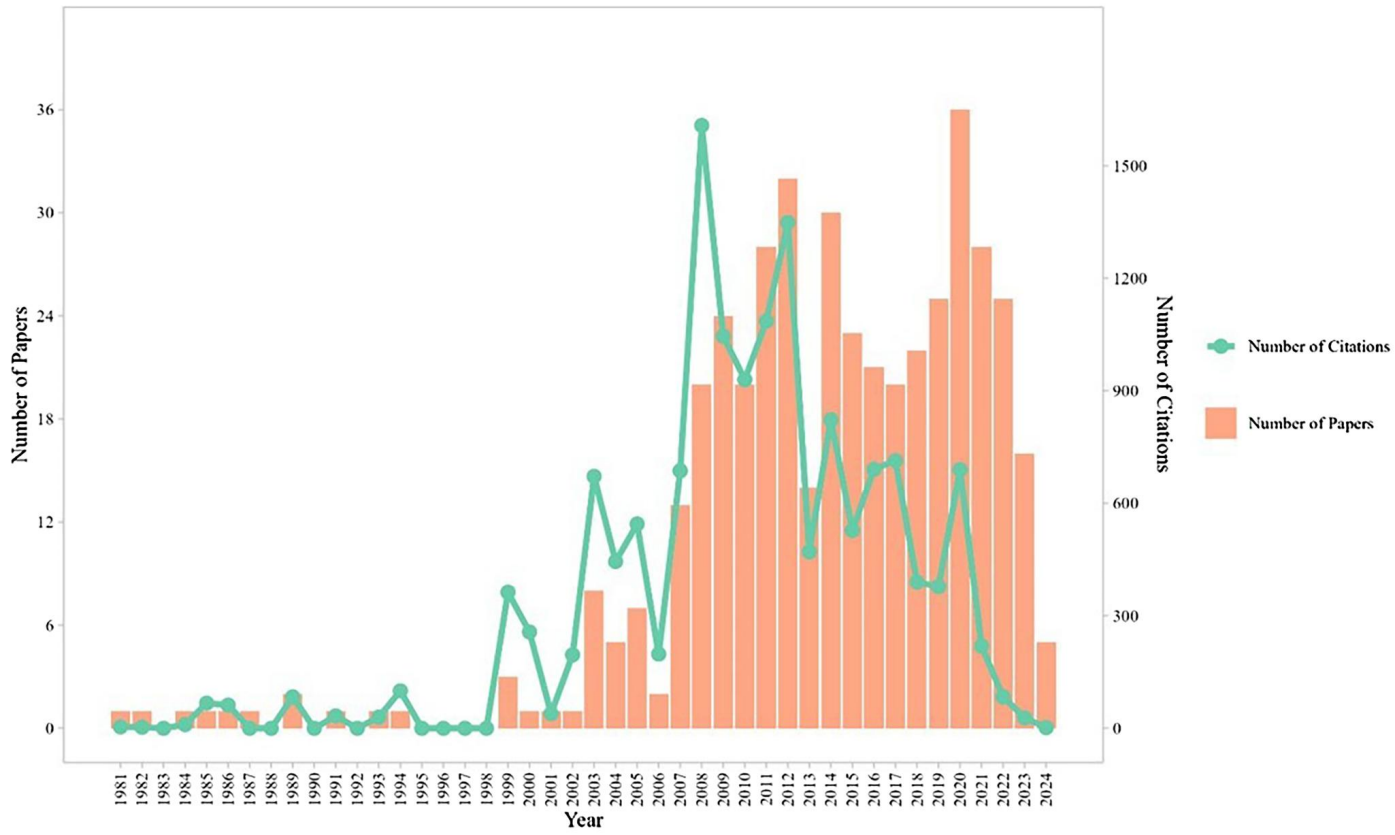
Numbers of papers and citations during study period. This figure, generated using VOSviewer (version 1.6.20) [9], displays the annual counts of published papers along with the total citations they have received over the study period.

Figure 1 shows the overall publication trend from 1981 onwards on the left axis. The number of research articles has generally increased over the years. Notably, in 14 different years, there were at least 20 publications. The highest number of papers published in a single year occurred in 2020, with 36 studies. On the right axis, the number of citations for the selected papers is displayed. The most citations for a single paper occurred in 2009, with 263 citations. Overall, the highest total citations for the selected papers were in 2008, with 1608 citations across 20 papers published that year. More than 60% of the citations are concentrated in the period from 2008 to 2017. The positive trends for citations continued until around 2017, after which there was a dramatic decrease in citations despite ongoing publication of new papers.

### Geographical distribution of author affiliations

The distribution in countries for the authors’ affiliations showed that Sweden has the highest frequency at 165, followed by Denmark with 124, Norway with 112, and Finland with 73 (data not shown).

### Co-authorship network of countries in research collaborations

**Figure 2.**
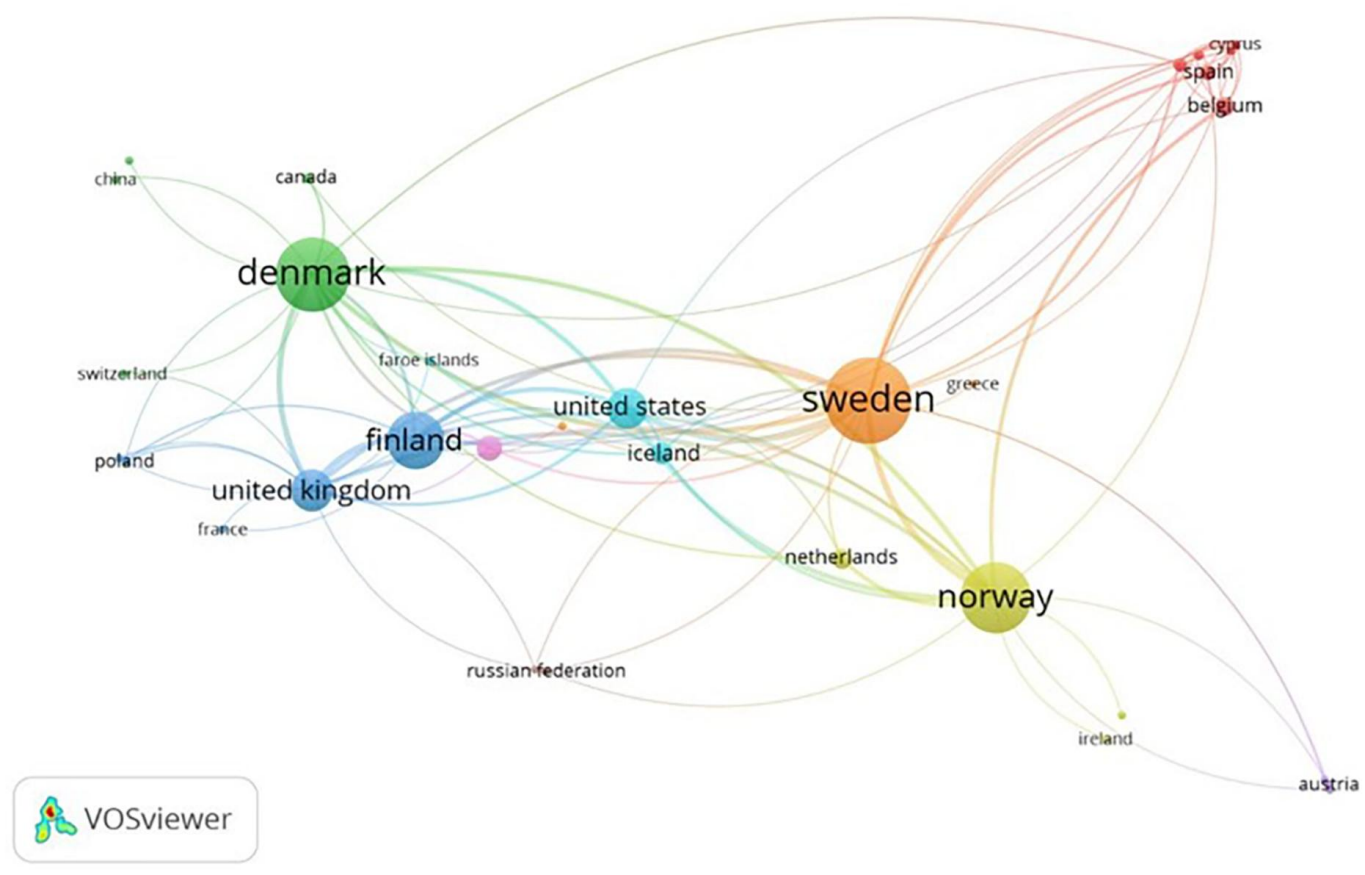
Co-authorship network of countries in research collaborations. The network diagram visualizes international research collaborations, where nodes represent countries, and the connection links indicate co-authorship links.

Figure 2 is the network visualization of co-authorship network, showing nodes representing Sweden, Denmark, and Norway as leading players in collaboration with other countries in research works. The dominant contributor is Sweden, which leads the list with a link strength of 42 and 1445 co-authored publications, showing strong links with Belgium at a link strength of 14, with Greece and the Netherlands. Denmark links at a link strength of 32, showing close collaborations with Finland at a link strength of 22 and showing links with both the United States and Canada at 4. Norway has a link strength of 25, with notable collaborations with Ireland and Austria at 4 each. Iceland is smaller but still has a link strength of 11, mainly in collaboration with the United States and Nordic countries. Also, Finland links well with Poland at 8 and with the United Kingdom at 10, underlining the powerful regional and international research partnerships of the Nordic countries.

### Bibliographic coupling network

The thematic connections between countries through shared citations showed that Denmark has the strongest presence, with a link strength of 4115, followed by Finland and Sweden, each with 3595. Belgium (3595) and Canada (944) are also well-connected, indicating substantial overlap in their cited literature. Austria (299) and China (140), though less connected, still show notable alignment with other countries (data not shown). Overall, this network reflects strong thematic ties, particularly among the Nordic countries, in the global research on childhood obesity.

### Subject area of publications

**Figure 3.**
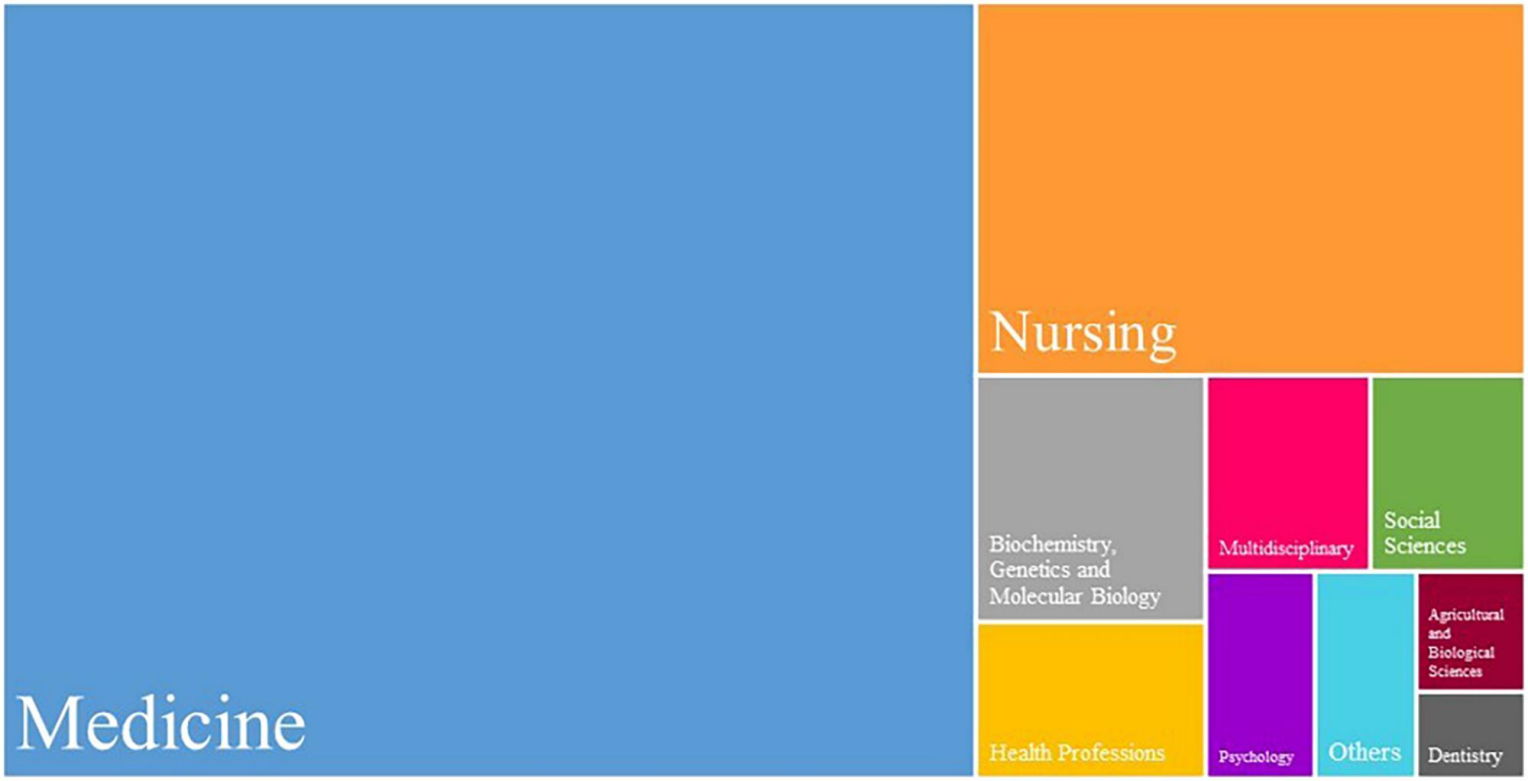
Distribution of documents by subject area. This figure, generated using VOSviewer (version 1.6.20) [9], presents the proportion of documents categorized into various subject areas.

The documents’ distribution into subject areas is shown in Fig. 3. Medical fields are dominant, as Medicine accounts for 64% out of the total 627 documents, followed by Nursing with 17%. Biochemistry, Genetics, and Molecular Biology make up 5% together, Health Professions 3%, and Multidisciplinary 3%. Other important contributions are Social Sciences, Psychology, and Agricultural and Biological Sciences with 3%, 2%, and 1%, respectively. Evidently, health-related research represents these top categories.

### Keywords co-occurrence

**Figure 4.**
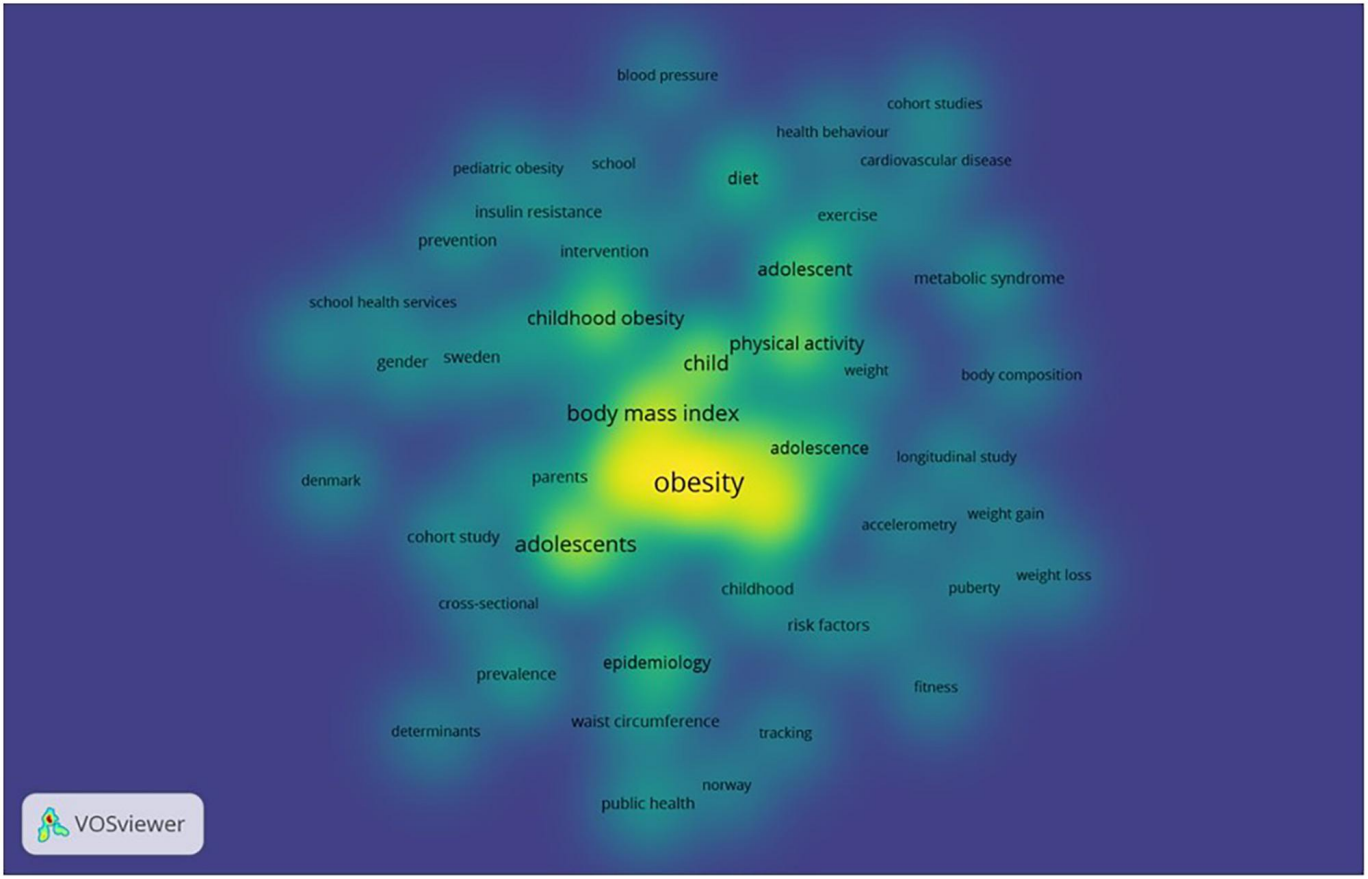
Network of co-occurring keywords with at least five occurrences. This figure visualizes the relationship among keywords that appears at least five times in the dataset.

Figure 4 shows the network visualization of co-occurring author keywords, limited to those with a minimum of five occurrences. Out of a total of 773 keywords, 55 met this threshold. “Obesity” appears most frequently, with 123 occurrences and a total link strength of 323. “Overweight” follows with 90 occurrences and a total link strength of 235. Other notable keywords include “children” with 78 occurrences (link strength: 182) and “body mass index” (BMI) with 58 occurrences (link strength: 154). “Blood pressure” has five occurrences, with the highest average citations (78.6).

### Institutional affiliations occurrences

Supplementary Fig. S2 presents the institutional affiliations occurring more than 20 times in the dataset. The results show that 161 institutes contributed to the papers and the total frequency of author affiliations are 1486. Karolinska Institute (Karolinska Institutet), Sweden has the highest frequency of affiliations, 77 times, followed by the University of Copenhagen (Københavns Universitet), Denmark with 72 affiliations. The faculty of health science, University of Copenhagen (Det Sundhedsvidenskabelige Fakultet, Københavns Universitet), Denmark and the Novo Nordisk Foundation Center for Basic Metabolic Research, University of Copenhagen, Denmark are each represented by 42 affiliations. Other major institutions are University of Southern Denmark (Syddansk Universitet) with 40, Gothenburg University (Göteborgs Universitet), Sweden with 39, and Copenhagen University Hospital with 36. Other major affiliating institutions include Helsinki (Helsingin Yliopisto), Finland with 35. The affiliating institutions shown here have had 20 or more affiliations to articles in Wikipedia.

### Institutional collaboration

Institutional collaboration with at least five documents published showed that Biocenter Oulu, University of Oulu, Finland participated with six documents. This institution received 257 citations, while its normalized citation score was 8.21. The University of Copenhagen, Faculty of Health and Medical Sciences contributed with ten documents, established four connections and had a total link strength of eight, accumulating 270 citations with a normalized citation score of 12.18. Finnish Institute of Occupational Health equally contributed six documents with three connections, a total link strength of 11, and 275 citations with a normalized citation score of 8.44. The last one was the Hans Christian Andersen Children’s Hospital, Odense university hospital, Denmark with six documents contributed, two connections, a total link strength of 3, 137 citations received, and a normalized citation score of 6.53 (data not shown).

## Discussion

The number of publications on childhood overweight/obesity within the Nordic countries has increased over the years, which indicates growing concerns about its public health complications in the region. The peak in the research output occurred in 2020, with 36 studies published. The most researched topic out of these 36 studies was health and diseases within the field of medicine (67%), followed by nursing (17%). However, this trend did not continue in the last 3 years. While childhood obesity gained significant attention during COVID-19 pandemic due to its adverse effects on diet and physical activity patterns in children [10, 11], the recent drop in publication might be due to increased focus shifts in research funding or priorities regarding health issues, which could influence the number of new studies being conducted and published in this field. Additionally, the focus on nursing research highlights the increased urgency of care practices in managing obesity during the pandemic.

Despite the growing research in recent years, the most cited paper, receiving 263 citations, was published by Norwegian researchers in 2009 [12]. This randomized trial explored the effect of a multidisciplinary approach and aerobic interval training on cardiovascular risk factors in adolescent with overweight or obesity. This research provided novel findings on reducing several known cardiovascular risk factors, including insulin, fasting glucose, etc. This may suggest that despite recent advancement this study continues to have enduring influence to shape ongoing research on strategies for reducing cardiovascular risks, even as much has changed since its publication.

Moreover, over 60% of all citations occurred in the period from 2008 and 2017. This indicates that much of the foundational research on childhood obesity within the Nordic countries was produced and gained recognition during that time. The pattern in the region aligns with global findings from a previous study [13]. Approximately 60% of publications worldwide on childhood obesity were produced between 2011 and 2017 [13]. While our finding covers a slightly broader period, the overlap in the timeframe suggests that in both global scale and the Nordic region, the early 2010s was a critical phase of research expansion in the field of childhood obesity. We believe that this period may reflect dietary transition and more urbanization, which contribute to more sedentary lifestyle among children. This association is well-documented in previous studies [14]. Additionally, it highlights the urgency of addressing childhood obesity as a multifactorial public health issue in this timeframe, which led to increased funding and policy focus.

Recent research from the Nordic countries seems to receive fewer citations, which may indicate that the current topics are perceived as less relevant by the academic community, or saturation in the field where new publications are not adding significant new knowledge. Therefore, we encourage researchers in the Nordic region to use health registers more effectively to conduct comprehensive studies. Additionally, focusing on studies that align with global trends, including emerging treatment options, pharmacotherapy, and integrated lifestyle interventions could enhance the relevance and impact of their work.

Geographical distribution of authors’ affiliations showed that researchers in Sweden are leading research related to childhood obesity in the region. Although the Nordic countries share common priorities in public health [15, 16], and even though they all have shifted the responsibility for obesity prevention interventions to the local municipality level in recent years [17], they do not seem to have a unified health policy on childhood obesity research. It might be due to different national perspectives on this issue. Therefore, we think that although the Nordic countries have similar prevalence rates of overweight and obesity [3, 6], Sweden has taken the lead, possibly due to the availability of more resources, funding and personnel, a strong network of researchers, and their institutions’ focus on this important public health issue. Hence, we call on both public and private institutions, as well as industry sectors to offer further funding opportunities for obesity research in the other parts of the Nordic region, as effective obesity management requires shared responsibilities and collaboration.

The bibliographic coupling network analysis showed Denmark’s strong leading position in producing foundational research that is widely cited by other countries. We believe that this could be due to a few factors, including novelty of the study, its methodological rigor, and strong findings derived from the rich and available registered data for the entire population. Additionally, the easy accessibility of the studies, such as through open access journals, plays a significant role [18] and likely contributes to the visibility and impact of a research within the international academic community.

The institutional affiliation analysis revealed that Karolinska university, Sweden and Copenhagen university, Denmark are at the forefront of research on childhood obesity driving research agendas and collaborative efforts across the globe. Other significant contributors include the faculty of health science, university of Copenhagen, Denmark and the Novo Nordisk foundation, center for basic metabolic research, university of Copenhagen, Denmark, which highlights the role of well-funded research-oriented organizations in advancing this field of study.

Citation scores of institutional collaborations revealed that University of Copenhagen, Faculty of Health and Medical Sciences not only contributed a significant amount of documentation but also achieved high citation rates. This suggest that their research is both influential and significant. We think that the current pattern of institutional collaboration emphasized that research impact is maximized when institutions exchange their expertise within the context of joint efforts.

The subject area distribution showed that research documents are preliminary centered in the medical and health sciences (64%). This reflects that primary focus of childhood obesity research in the Nordic region are interventions, treatment (e.g. bariatric surgery), and health outcomes. Notably, studies on the use of anti-obesity medication are underrepresented. Meanwhile, the American Academy of Pediatrics now emphasizes that a passive approach is insufficient to address the long-term impact of childhood obesity and recommends an early active use of anti-obesity medications to enhance treatment effectiveness [19]. Therefore, we invite researchers in the Nordic countries to explore this area, as it could potentially expand the treatment options available for better childhood obesity management, especially for children who struggle to achieve weight loss through lifestyle changes alone. The current presence of nursing research (17%) in addressing childhood obesity in areas like patients care and community health interventions highlights the importance of multidisciplinary approaches in tackling childhood obesity in the Nordic region. The interest in understanding the genetic and molecular mechanism was evidenced by only the 5% contribution of biochemistry, genetics and molecular biology in childhood obesity research. We believe that further investigating of this field is crucial, as they can reveal molecular pathways and genetic susceptibility involved in obesity. This knowledge could contribute to development of targeted interventions and treatment including anti-obesity medications for children [20]. A smaller but still relevant contribution was observed from social science (3%), psychology (2%), and agriculture and biological sciences (1%). This reflects that the Nordic countries are addressing obesity as a multifactorial health issue that goes beyond just medical and biological interventions. For instance, research in agriculture and biological sciences may focus on how agricultural practices, food availability, and nutrition policies impact children’s eating habit and consequently health outcomes. We believe this kind of information is essential to developing more effective prevention and intervention strategies.

The co-occurrence of keywords such as obesity, overweight, and children shows that much of the research is centered on defining and addressing obesity in pediatric populations, with attention to the metrics such as BMI. The significant link strength associated with these keywords emphasizes that they are not only frequently studied but also are highly interconnected within the literature. Previous studies indicated that BMI varies with age in children [21, 22]. Several studies have suggested using a comprehensive approach to evaluate metabolic status using BMI, waist-to-hip ratio, and other metabolic indicators that will enhance the assessment of obesity risk [23, 24]. Therefore, we advise researchers in the Nordic countries not to rely solely on BMI as it might not provide a clear picture of an individual’s health status.

Moreover, the key word “blood pressure” despite its low frequency stands out, because it has the highest average citations. This reflects that research focusing on childhood obesity and blood pressure is receiving significant attention in the region. This may reflect growing concern about the cardiovascular risks associated with childhood obesity, as elevated blood pressure in children can be an early indicator of future cardiovascular diseases [25, 26]. Additionally, in our view the high citation count indicates that this is a critical area of research with potentially significant implications for public health.

### Strengthens, limitations

Our study offers valuable insights into the childhood obesity research in the Nordic region for better research planning, and allocating health resources; however, limitations exist. Citation-based metrics may overemphasize studies in high-impact, English-language journals. This may distort the perceived influence of certain studies. Additionally, the databases used in this review may not provide a comprehensive view of emerging research areas found in non-indexed journals, particularly in the Nordic region, where publications in Nordic languages may be underrepresented.

### Future research

Future research should prioritize underrepresented areas like biochemistry, genetics, and social science to better understand childhood obesity, particularly its link to blood pressure. The authors believe that the unique datasets available in the Nordic countries, including large and complex national registers, provide a great opportunity to apply advanced analytical methods, such as machine learning, to enhance the prediction of obesity-related risks and outcomes. Moreover, additional research into pharmacotherapy for pediatric obesity is crucial to fulfill the existing treatment gap, particularly for children who are unable to achieve weight loss goals via lifestyle changes alone.

## Conclusions

Sweden is playing a prominent role in Nordic childhood obesity research, but in general the field remains highly collaborative and international. Regarding subject area, substantial progress has been made in understanding childhood obesity, with a dominant focus on medicine and nursing. Key research themes such as overweight, obesity, and BMI have been extensively explored, while blood pressure, despite appearing less often, has the highest citation count. This indicates growing interest in understanding the association between blood pressure and obesity among our target population in the Nordic region.

## Supplementary Material

ckaf053_Supplementary_Data

## Data Availability

No new data were gene or analyzed in support of this research.
