# Supplementary material for: Trends and future directions in childhood obesity research in the Nordic countries: a scientometric review
Source: Eur J Public Health. 2025 Apr 15;35(4):738–44. doi: 10.1093/eurpub/ckaf053 (PMC12311353; doi:10.1093/eurpub/ckaf053)
Supplement: ckaf053_Supplementary_Data [file ckaf053_supplementary_data.zip › ckaf053_Supplementary_Data/ejph-2024-12-om-0880-File006.docx]

**Table S 1:** Search strategy

This table outlines the detailed search strategy used for our scientometric review. It includes all search terms, Boolean operators, and limits applied across the database to identify relevant studies on Trends and Future Directions in Childhood Obesity Research in the Nordic-Countries.

| **Search domains** |
| --- |
| ( ( TITLE-ABS-KEY ( "Body Mass Index" )  OR  TITLE-ABS-KEY ( "BMI" )  OR  TITLE-ABS-KEY ( obes* )  OR  TITLE-ABS-KEY ( overweight )  OR  TITLE-ABS-KEY ( "Waist Circumference" )  OR  TITLE-ABS-KEY ( "WhtR" )  OR  TITLE-ABS-KEY ( "body fat" )  OR  TITLE-ABS-KEY ( "fat pattern" )  OR  TITLE-ABS-KEY ( "WC" ) ) )  AND  ( ( TITLE-ABS-KEY ( "School-age child" )  OR  TITLE-ABS-KEY ( child* )  OR  TITLE-ABS-KEY ( teen )  OR  TITLE-ABS-KEY ( adolescen* )  OR  TITLE-ABS-KEY ( " 6 to 18 years" )  OR  TITLE-ABS-KEY ( "6 – 18 years" ) ) |
| **Geographical domains** |
| ((TITLE-ABS-KEY (sweden ) OR TITLE-ABS-KEY ( finland ) OR TITLE-ABS-KEY ( iceland ) OR TITLE-ABS-KEY ( norway ) OR TITLE-ABS-KEY ( denmark ) OR TITLE-ABS-KEY ( "Nordic Countries" ) ) |
