# Supplementary material for: Trends and future directions in childhood obesity research in the Nordic countries: a scientometric review
Source: Eur J Public Health. 2025 Apr 15;35(4):738–44. doi: 10.1093/eurpub/ckaf053 (PMC12311353; doi:10.1093/eurpub/ckaf053)
Supplement: ckaf053_Supplementary_Data [file ckaf053_supplementary_data.zip › ckaf053_Supplementary_Data/ejph-2024-12-om-0880-File007.docx]

## Identification

Duplicates removed

(n =1,185)

Records identified through database searching (n =4,123)

## Screening

Records excluded as they do not meet selection criteria
(n =2,441)

Records screened
(n =2,938)

Full-text articles excluded with reasons
(n =56)

Full-text articles assessed for eligibility

(n =497)

## Eligibility

Studies eligible for analysis

(n =441)

## Included

Studies included in analysis

(n =441)

**Figure S1:** overview of the study selection process

This flow diagram outlines the review process for our scientometric review. It details the initial identification of the records through database search and other sources, followed by the removal of duplicates, screening of titles and abstracts, full-text assessment and final inclusion of studies in the review.
