# Supplementary material for: Trends and future directions in childhood obesity research in the Nordic countries: a scientometric review
Source: Eur J Public Health. 2025 Apr 15;35(4):738–44. doi: 10.1093/eurpub/ckaf053 (PMC12311353; doi:10.1093/eurpub/ckaf053)
Supplement: ckaf053_Supplementary_Data [file ckaf053_supplementary_data.zip › ckaf053_Supplementary_Data/ejph-2024-12-om-0880-File008.docx]

**Figure S2:** Institutional affiliations with more than 20 occurrences in the dataset

This figure presents the institutional affiliations that appear more than 20 times in the dataset. It highlights the most frequently represented institutions among the included studies, offering insights into the concentration of research output from these organizations.
